# Long non‐coding RNA SNHG1 stimulates ovarian cancer progression by modulating expression of miR‐454 and ZEB1

**DOI:** 10.1002/1878-0261.12932

**Published:** 2021-03-19

**Authors:** YinYing Wu, Bo Zhu, Yanli Yan, Shuheng Bai, Haojing Kang, Jiangzhou Zhang, Wen Ma, Ying Gao, Beina Hui, Rong Li, Xiaozhi Zhang, Juan Ren

**Affiliations:** ^1^ Department of Chemotherapy, Oncology Department First Affiliated Hospital of Xi’an Jiaotong University China; ^2^ Department of Pulmonary and Critical Care Medicine First Affiliated Hospital of Xi’an Jiaotong University China; ^3^ Department of Radiotherapy, Oncology Department First Affiliated Hospital of Xi’an Jiaotong University China; ^4^ Medical School Xi’an Jiaotong University China

**Keywords:** microRNA‐454, migration, ovarian cancer, small nucleolar RNA host gene 1, zinc finger E‐box‐binding homeobox 1

## Abstract

Ovarian cancer (OC) is highly prevalent and is associated with high mortality rates due to metastasis and relapse. In this study, we assessed the role of long non‐coding RNA (lncRNA) small nucleolar RNA host gene 1 (*SNHG1*) in OC to gain further insight into mechanisms that contribute to its aggressiveness. We analyzed the correlation between *SNHG1*, *miR‐454* and zinc finger E‐box‐binding homeobox 1 (*ZEB1*) using a dual‐luciferase reporter assay. Alterations in cell metastasis and invasiveness were observed using wound‐healing and Transwell invasion assays, respectively. Tumor xenografts allowed us to monitor liver metastasis of mice injected with A2780 cells. We found that *SNHG1* is overexpressed in OC. Downregulation of *SNHG1* promoted *miR‐454* expression and reduced *ZEB1* levels. In addition, knockdown of *SNHG1*, also reduced the aggressiveness of A2780 and SK‐OV3 cells. Furthermore, *SNHG1* downregulation by siRNA hindered cell migration and invasion; however, this effect was reversed by co‐transfection of *miR‐454* into A2780 and SK‐OV3 cells. Moreover, *SNHG1* increased *ZEB1* expression by downregulating *miR‐454* and activated Akt signaling, thereby promoting epithelial‐mesenchymal transition and enhancing the invasiveness of OC cells. Tumor xenograft analyses confirmed that *SNHG1* affects OC proliferation and metastasis *in vivo*. In summary, our data demonstrate that *SNHG1* plays crucial roles in tumor progression and may be a useful maker for OC prognosis.

## Graphical abstract

LncRNA SNHG1 promotes proliferation and migration of ovarian cancer cells through the sponging of miR‐454 and activation of the PI3K/AKT pathway, respectively. MiR‐454 was found to inhibit zinc finger E‐box‐binding homeobox 1 (ZEB1) expression and function by directly targeting the 3’‐UTR of ZEB1.

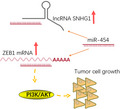

**Abbreviations**
ANOVA: analysis of variance
EMT: epithelial‐mesenchymal transition
H&E: hematoxylin and eosin
lncRNA: long non‐coding RNA
miRNA: microRNA
OC: ovarian cancer
PDAC: pancreatic ductal adenocarcinoma
qRT‐PCR: quantitative real‐time polymerase chain reaction
SNHG1: small nucleolar RNA host gene 1
TNBC: triple‐negative breast cancer
TNM: tumor node‐metastasis
ZEB1: zinc finger E‐box‐binding homeobox 1

## 1 Introduction

Ovarian cancer (OC) is the seventh most prevalent cancer and a major source of cancer‐associated mortality in women, due to metastasis and relapse [1]. In spite of advances in surgical procedures and chemotherapeutics, the overall 5‐year survival rates in OC cases are still below 30% [2]. Tumor invasion, metastasis and chemoresistance play critical roles in the poor prognosis associated with OC [3]. Even though the invasiveness and metastasis of cancer cells are regulated by several cellular and signaling proteins, the development of these abilities by tumors is associated with the diminution of epithelial characteristics and attainment of mesenchymal traits, a phenomenon termed epithelial‐mesenchymal transition (EMT) [4, 5]. Consequently, it is essential to identify factors that inhibit EMT as well as to comprehend the underlying mechanisms for the development of effective treatments for OC.

Long non‐coding RNA (lncRNA) are transcriptional nucleic acids larger than 200 nucleotides that lack coding functions [6, 7]. Numerous lncRNA have been established to be connected to tumor development [8, 9]. Additionally, earlier studies have shown that the lncRNA expressions differed considerably between OC tissues and healthy ovarian tissues [7, 10]. Overexpression of small nucleolar RNA host gene 1 (SNHG1), an lncRNA, is linked to the proliferation of gastric tumor cells [11], whereas the downregulation suppressed colorectal carcinogenesis [12]. In addition, SNHG1 encouraged proliferation in glioma [13], prostate cancer [14], non‐small‐cell lung carcinoma [13], etc. Nevertheless, only a few investigations on the functional importance of lncRNA in OC have been reported.

MicroRNA (miRNA) are short non‐coding RNA that hinder the translation of their target mRNA [15]. It was established that dysregulated miRNA are involved in several cancers, but the function of specific miRNA is unclear [16]. A few miRNA play vital roles in the diagnosis and treatment of cancer [17]. For instance, miR‐454 inhibited the tumorigenesis and development of pancreatic ductal adenocarcinoma (PDAC) [18]. In addition, miR‐454 expression was closely correlated with tumor metastasis to chondrosarcoma of the lymph nodes [19]. However, a high proportion of miR‐454 was linked to the poor prognosis of triple‐negative breast cancer (TNBC) [20].

In this study, how SNHG1 influences OC development was investigated. We have hypothesized that SNHG1 may modulate zinc finger E‐box‐binding homeobox 1 (ZEB1) expression through miR‐454 and thereby control the progression OC. In this context, this study could be helpful for the development of therapeutic strategies against OC.

## 2 Methods

### 2.1 Tissue specimens

Tumor tissues and neighboring healthy tissues were collected surgically from 30 OC patients who had not received any other treatments, in the First Affiliated Hospital of Xi’an Jiaotong University from February 2011 to November 2015. The tissues were differentiated based on the tumor node‐metastasis staging system. Following resection, the tissues were preserved at −80 °C. This study was conducted according to the principles expressed in the Declaration of Helsinki. This investigation was authorized by the Ethics Committee of Xi’an Jiaotong University and informed assent was obtained from all patients.

### 2.2 Cell culture

Human OC cell lines A2780, OCC1, H8710 and SK‐OV3 as well as the normal cell line HMEC‐1 were acquired from the Cell Bank of the Chinese Academy of Sciences. They have been grown in Dulbecco’s modified Eagle media (DMEM) that also contains 1% penicillin/streptomycin and 10% FBS and were cultured in a humidified 5% CO_2_ environment at 37 °C.

### 2.3 Cell transfection

Cells were inoculated into six‐well plates and transfected with miR‐454 mimics, inhibitor (miR‐454‐in), or Negative Control (NC) by means of Lipofectamine 2000 (Invitrogen, Carlsbad, CA, USA). The partial sequences employed in this investigation are presented in the Table S1.

### 2.4 Quantitative real‐time PCR

MicroRNA and mRNA were isolated by means of the mirVana miRNA isolation kit and the TRIzol technique, respectively. Total RNA (1 µg) was reverse‐transcribed into cDNA by means of TaKaRa, and cDNA (50 ng) was used for each reaction. Quantitative real‐time PCR (qRT‐PCR) was performed by means of SYBR® Green PCR Master Mix on an ABI Prism 9700 Sequence Detection System (Applied Biosystems). The primer sequences employed for qRT‐PCR are displayed in Table S2. Samples were assayed in duplicate. Samples were normalized using U6 small nuclear RNA, and the comparative expressions were determined by means of the 2^−ΔCt^ technique.

### 2.5 Western blotting

Cells were cultured for 24 h in six‐well plates (5 × 10^5^ cells per plate). The culture medium was removed, cells washed using PBS and treated with RIPA lysis buffer comprised of proteinase inhibitor (Sigma, St.Louis, MO, USA) for 30 min. Proteins were collected from the lysed tissues by centrifuging for 20 min at 12 000 ***g*** and the total amount of protein quantified via BCA assay (Pierce, Rockford, IL, USA). After running on a 10% SDS/PAGE gel, protein was transferred to a polyvinylidene difluoride membrane and 5% non‐fat milk was used for a 2‐h blocking step. Further, they were incubated with primary antibodies against E‐cadherin, N‐cadherin and MMP‐2 (1 : 1000; Cell Signaling Technology, Danvers, MA, USA) as well as ZO‐1, Vimentin and MMP‐9 (1 : 1000; Abcam, Cambridge, MA, USA) at 4 °C overnight followed by rinsing and incubating for 2 h with the respective horseradish peroxidase‐labeled secondary antibodies (1 : 5000; Santa Cruz, Santa Cruz, CA). The protein bands were visualized by means of enhanced chemiluminescence (ECL) kits (Abcam). GAPDH was used as a loading control.

### 2.6 Cell viability assay

Transfected cells were inoculated into 96‐well plates (1500 cells per well) for 24 h and the cell viability test was conducted using Cell Counting Kit‐8 (Dojindo, Kumamoto, Japan) based on the supplier’s instructions. The absorbance at 450 nm was calculated. Experiments were carried out in triplicate.

### 2.7 Wound‐healing assay

Wound‐healing assay was carried out to study the cell metastatic ability. In particular, cells were cultured until 90–95% confluence in six‐well plates on which wounds were made with a 10‐μL pipette tip. Cells were rinsed with serum‐free DMEM and photographed to document the wound widths at 0 h. After 24 h, the wound widths were photographed for a second time at specific times and the cell migration was determined by measuring the gaps in multiple fields.

### 2.8 Cell invasion assay

Invasiveness of the cells was determined by means of the cell invasion assay following the supplier’s directions (BD Biosciences, San Jose, CA, USA). After 24 h, cells were treated with 95% ethanol and 0.1% crystal violet, and enumerated using a light microscope. The mean number of invaded cells was obtained by averaging the number of cells of 10 fields in two inserts. Experiments were carried out in duplicate and repeated at least three times.

### 2.9 Luciferase activity assays

The wild‐type (wt) and mutated (mut) sequences of SNHG1 possessing the miR‐454 recognition site were cloned into psiCHECK2 to obtain the recombinants wt‐SNHG1 and mut‐SNHG1, respectively. The wt and mut sequences of ZEB1 3’‐UTR cDNA possessing the miR‐454 active site have been cloned into the psiCHECK2 to obtain the recombinants wt‐ZEB1 and mut‐ZEB1, respectively. HEK293T cells were co‐transfected with recombinant plasmids (100 ng) and miR‐454‐ or miR‐NC‐mimics (50 pmol). After 48 h, their luciferase activity has been calculated by means of the Dual‐Luciferase Reporter Assay system (Promega, Beijing, China).

### 2.10 Animal experiments

Fourteen female BALB/c nude mice (6 weeks old) were bought from Shanghai Experimental Animal Center of the Chinese Academy of Sciences. The experiments were authorized by the Animal Experimental Ethics Committee of Xi’an Jiaotong University. To establish a xenograft tumor model, 1  × 10^6^ A2780 cells transfected with siSNHG1 or shCON per 150 µL PBS were subcutaneously injected (*n* = 7/group, 14 in total) and tumor nodules were gauged every 2 days. In the meantime, bodyweight, skin color and mobility were monitored. Tumor volume was estimated using the formula: Volume = 1/2 × length × width^2^. Diminished mobility was seen in the mice of the A2780 control group but no mouse died. After 24 days, mice were euthanized by CO_2_ asphyxiation and the tumors were excised, measured, photographed and pathologically investigated.

### 2.11 HE staining

Tissues were deparaffinized twice in xylene for 10 min. They were then immersed in diminishing concentrations of ethanol (100%, 95%, 80%, 70% and 50%) for 2 min each. They were stained in hematoxylin for 5 min and treated with 1% HCl‐alcohol for 20 s, followed by washing under tap water for 2 min. Subsequent to dehydrating in increasing concentrations of ethanol (50%, 70% and 80%), tissues were subjected to 1% NH_4_OH and eosin for 2 min, followed by 95%, 100% or 100% ethanol for 2 min each. Finally, tissues were cleared in xylene and mounted in a resin.

### 2.12 Statistical analysis

Data are given as means ± standard deviation (SD). The software used was spss 24.0 (IBM, Armonk, NY, USA) and graphpad prism 6.0 (Graphpad software, San Diego, CA, USA). Student’s *t*‐test was used for two dataset comparisons, and one‐way analysis of variance (ANOVA for those involving more than three sets. *P* < 0.05 was taken as the threshold for statistical significance.

## 3 Results

### 3.1 MiR‐454 is downregulated in OC tissues

**Fig. 1:**
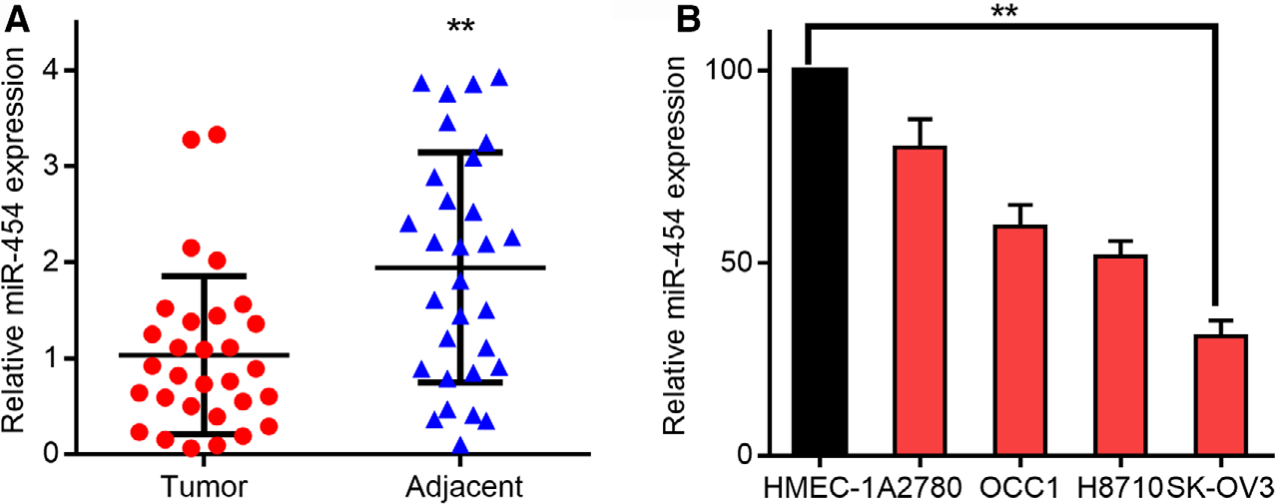
MiR‐454 expression in OC and neighboring tissues. Relative miR‐454 levels in (A) OC and neighboring tissues and (B) HMEC‐1, A2780, H8710, SK‐OV3 and OCC1 cell lines. Tumor (*n* = 30), adjacent (*n* = 30). The measurement data are expressed as mean ± standard deviation. Comparisons between two groups are analyzed using non‐paired *t*‐tests (A). Comparisons among multiple groups were analyzed using one‐way ANOVA (B). **P < 0.01.

To monitor the expression of miR‐454 in OC, qRT‐PCR analysis was carried out using 30 pairs of human primary OC and neighboring tissues. In OC tissues, miR‐454 expression was considerably lower relative to pair‐matched neighboring tissues (**P < 0.01, Fig. 1A). These outcomes indicated that miR‐454 downregulation correlated positively with the advancement of OC. Further, we explored whether miR‐454 level was similarly correlated in the following ovarian cell lines: HMEC‐1, A2780, H8710, SK‐OV3 and OCC1. Indeed, miR‐454 was downregulated in OC cell lines relative to the normal control (Fig. 1B). Among these OC cell lines, miR‐454 was upregulated in A2780, which was used for the subsequent test. In contrast, miR‐454, expressed at a low‐level in the SK‐OV3, was upregulated in the miR‐454‐overexpression test. Collectively, these data signified that miR‐454 downregulation appreciably correlated with the proliferation of OC.

### 3.2 MiR‐454 overexpression hinders development, motility and invasiveness of OC cells

**Fig. 2:**
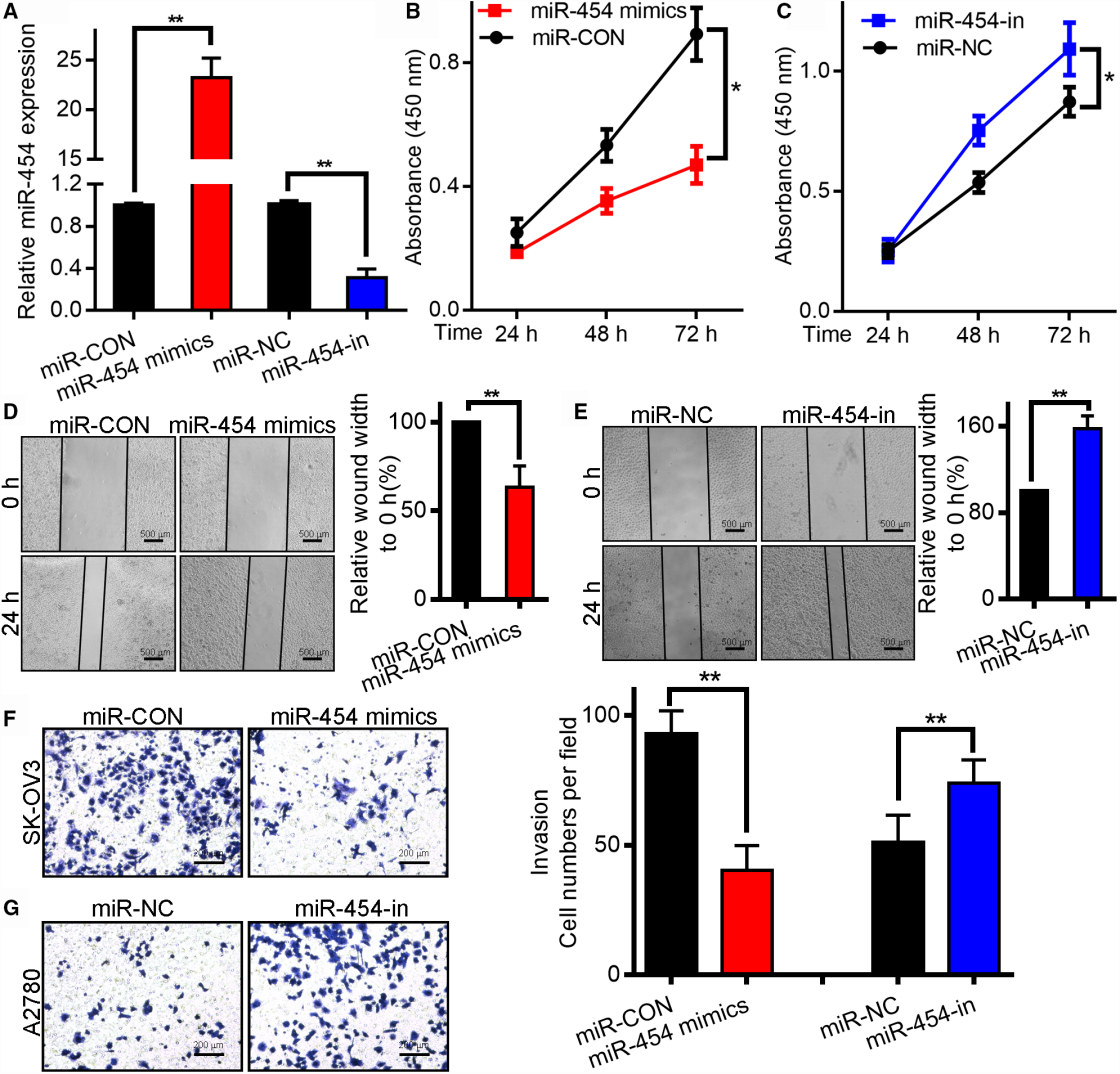
MiR‐454 modulated the tumorigenesis and invasiveness of OC cell lines. (A) The efficiency of miR‐454 mimic‐transfection into SK‐OV3 cell line was determined through qRT‐PCR. ** vs miR‐CON mimics, *P* < 0.01. The effectiveness of miR‐454‐in‐transfected into A2780 cells was determined by qRT‐PCR. ** vs inhibitor miR‐NC, *P* < 0.01. (B) The proliferation of SK‐OV3 cells was decreased after miR‐454 overexpression. (C) The growth of A2780 cells was enhanced subsequent to the knockdown of miR‐454. (D, E) Wound‐healing study of SK‐OV3 and A2780 cells expressing mimic or inhibitor. Scale bar: 500 μm. (F,G) Impact of miR‐454 overexpression or knockdown on the invasiveness of SK‐OV3 and A2780 cells was studied via Transwell assays. Scale bar: 200 μm. The measurement data are expressed as mean ± standard deviation. Comparisons between two groups were analyzed using non‐paired *t*‐tests. * vs miR‐NC or miR‐CON, *P* < 0.05. ** vs miR‐NC or miR‐CON, *P* < 0.01.

The impact of miR‐454 on the tumorigenesis and progress of OC was investigated in OC cell lines through the use of its inhibitor as well as mimics. The level of miR‐454‐in was reduced in the A2780 cell line relative to the miR‐NC group, whereas the level was amplified by miR‐454 mimics in SK‐OV3 cells relative to the miR‐CON mimic group (Fig. 2A). When the SK‐OV3 cells were transfected with miR‐454, cell proliferation decreased drastically after 72 h (Fig. 2B). Furthermore, the wound‐healing study confirmed that higher expression levels of miR‐454 reduced the metastasis of SK‐OV3 cells compared with miR‐CON control cells (Fig. 2D), whereas its knockdown accelerated the metastasis of A2780 cells (Figs. 2C,E). Transwell assays established that miR‐454 overexpression hindered the invasiveness of SK‐OV3 cells relative to miR‐CON control cells (Fig. 2F), whereas its inhibition stimulated the invasiveness of A2780 cells (Fig. 2G). These results indicate that miR‐454 impairs proliferation, metastasis and invasiveness.

### 3.3 MiR‐454 modulates EMT via PI3K/Akt cascade

**Fig. 3:**
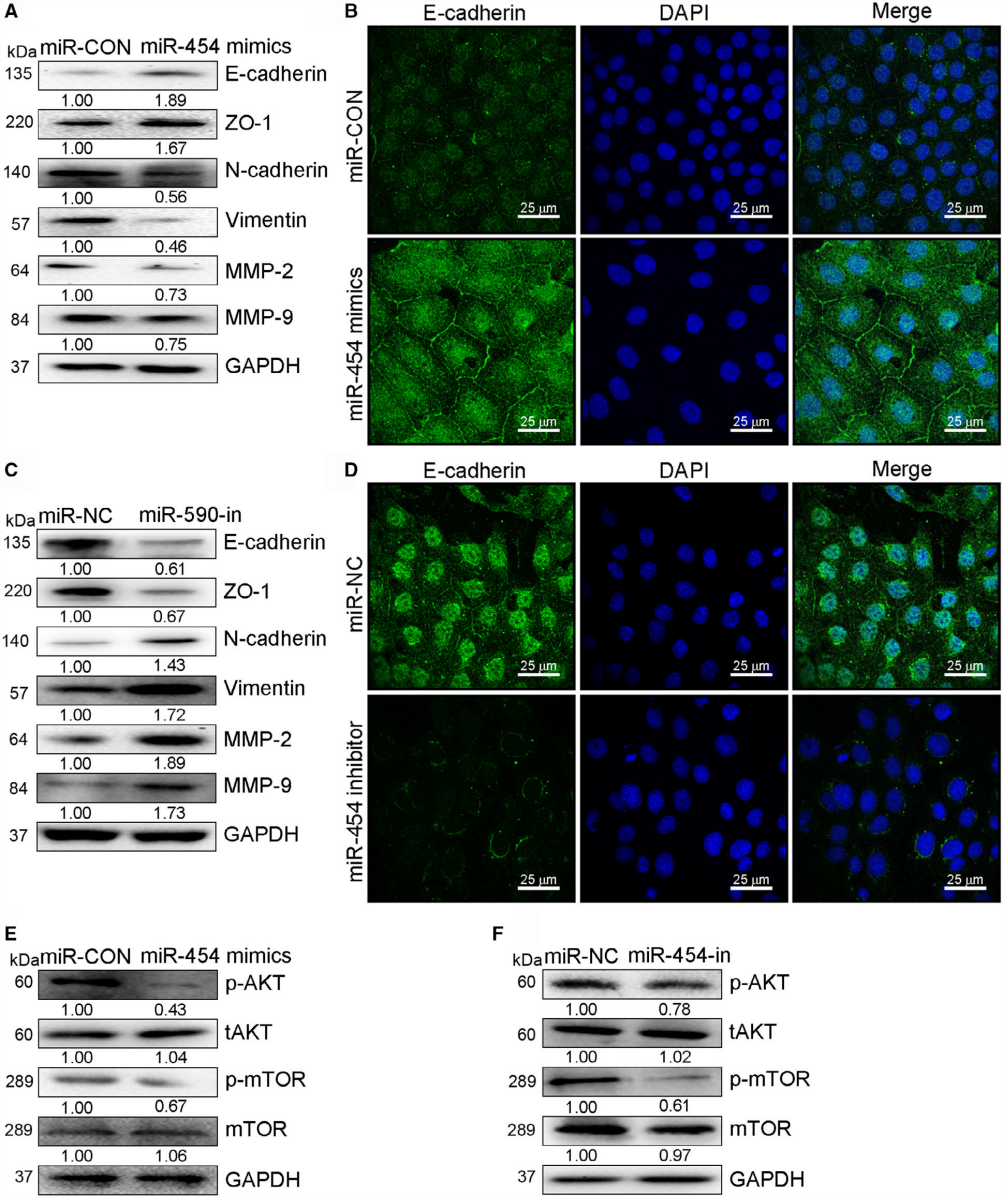
MiR‐454 inhibited OC cell invasiveness and EMT by inactivating Akt signaling. (A) The protein expressions of ZO‐1, N‐cadherin, MMP‐2, MMP‐9, vimentin, and E‐cadherin in SK‐OV3 cells. (B) Immunofluorescence study of the expression of E‐cadherin in SK‐OV3 cells transfected with miR‐CON or mimics. Scale bar: 25 μm. (C) The protein expressions of ZO‐1, N‐cadherin, MMP‐2, MMP‐9, vimentin, and E‐cadherin in A2780 cells. (D) Immunofluorescence study of the expression of E‐cadherin in miR‐NC‐ or miR‐454‐transfected A2780 cells. Scale bar: 25 μm. (E,F) Western blot measurement of phosphorylated (active) Akt and mTOR in OC cells upon transfection of mimic or miR‐454‐in.

E‐cadherin is an epithelial marker, whereas MMP‐2 and ‐9 are mesenchymal markers. Since their changes correlate with EMT [21], the expressions of these markers (ZO‐1, N‐cadherin, vimentin and E‐cadherin), MMP‐2 and ‐9 were studied in SK‐OV3 and A2780 cell lines. The expression of N‐cadherin, vimentin, MMP‐2, and MMP‐9 in SK‐OV3 cell line was reduced following miR‐454 overexpression, whereas that of E‐cadherin and ZO‐1 was amplified. An opposite effect was observed in miR‐454‐in‐transfected A2780 cells (Fig. 3A,C). The immunofluorescence assays showed the same results (Fig. 3B,D). With respect to the Akt signaling, miR‐454 overexpression reduced the expression of phosphorylated Akt and mTOR in SK‐OV3 cells, whereas miR‐454 knockdown augmented the expression in A2780 cells (Fig. 3E,F).

### 3.4 ZEB1 is a miR‐454 target in OC

**Fig. 4:**
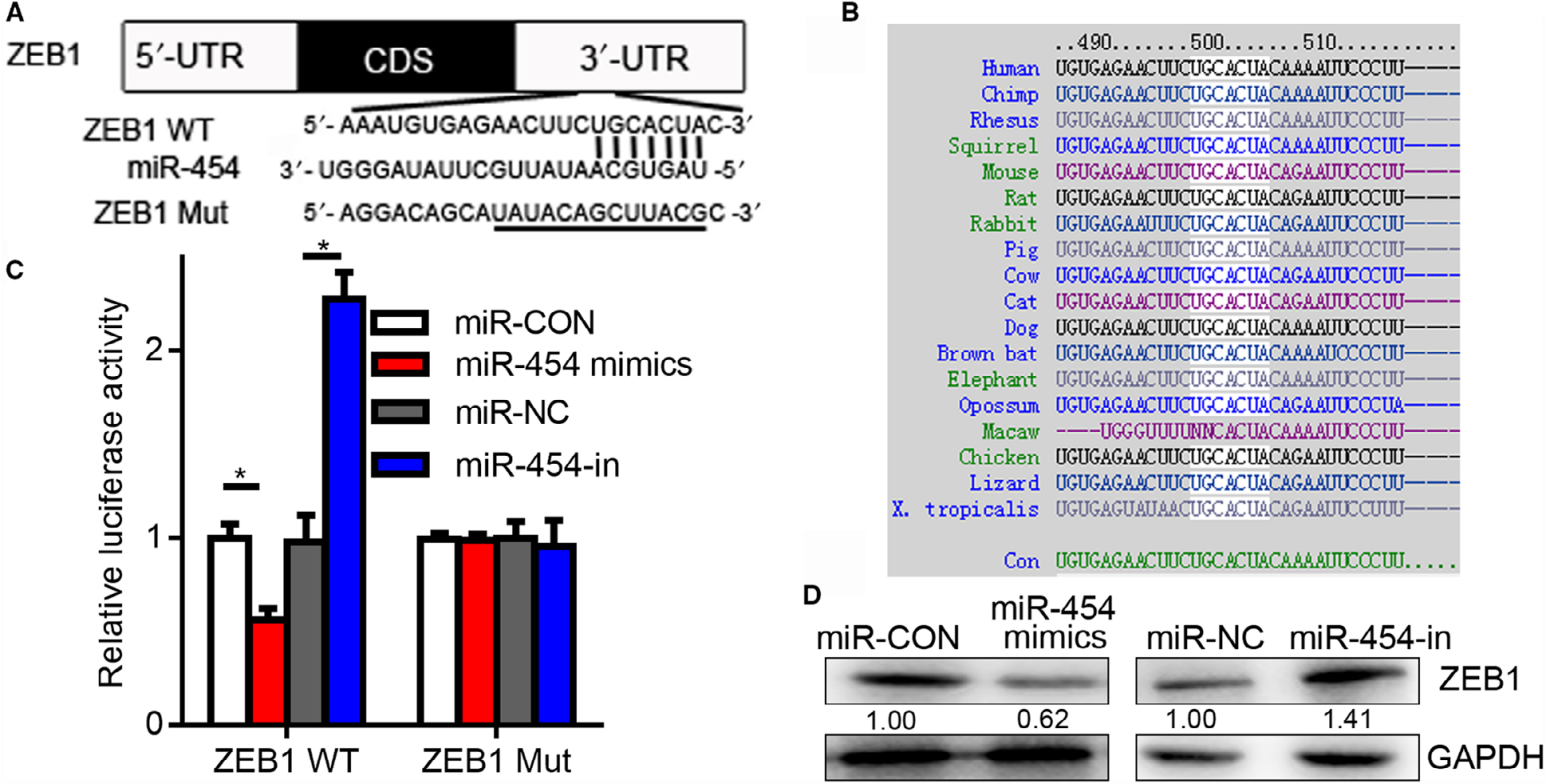
ZEB1 is a direct miR‐454 target. (A) MiR‐454 putative and mutated binding sites in the 3’‐UTR of ZEB1. (B) Base pairing between miR and target sequences of ZEB1. Conserved sequences are shown across species (white). (C) Impact of miR‐454 on the level of ZEB1 3’‐UTR‐containing reporter genes. ** vs miR‐NC or miR‐CON, *P < 0.05. (D) Protein expression of ZEB1 in miR‐454 mimic‐ or inhibitor‐transfected OC cells. mut, mutant. Measurement data are expressed as mean ± standard deviation. Comparisons among multiple groups were analyzed using one‐way ANOVA.

The binding sites between ZEB1 and miRNA were revealed through target gene prediction analysis. ZEB1, a transcription factor, is overexpressed in several human cancers, including OC [22]. Jin *et al*. [23] established that in OC, induction of ZEB1 can stimulate the PI3K/Akt pathway. Furthermore, inhibition of ZEB1 downregulates the constitutive phosphorylation of Akt in numerous cell lines [24, 25]. ZEB1 was therefore selected for further investigations (Fig. 4A). The miR‐454 mimic abolished wt‐ZEB1 luciferase activity (Fig. 4B), whereas miR‐454‐in enhanced it (Fig. 4C). In addition, the mimic notably reduced ZEB1 expression, although miR‐454‐in amplified ZEB1 expression (Fig. 4D). All these results indicate that ZEB1 is a miR‐454 target gene.

### 3.5 Reciprocal repression between SNHG1 and miR‐454 in OC

**Fig. 5:**
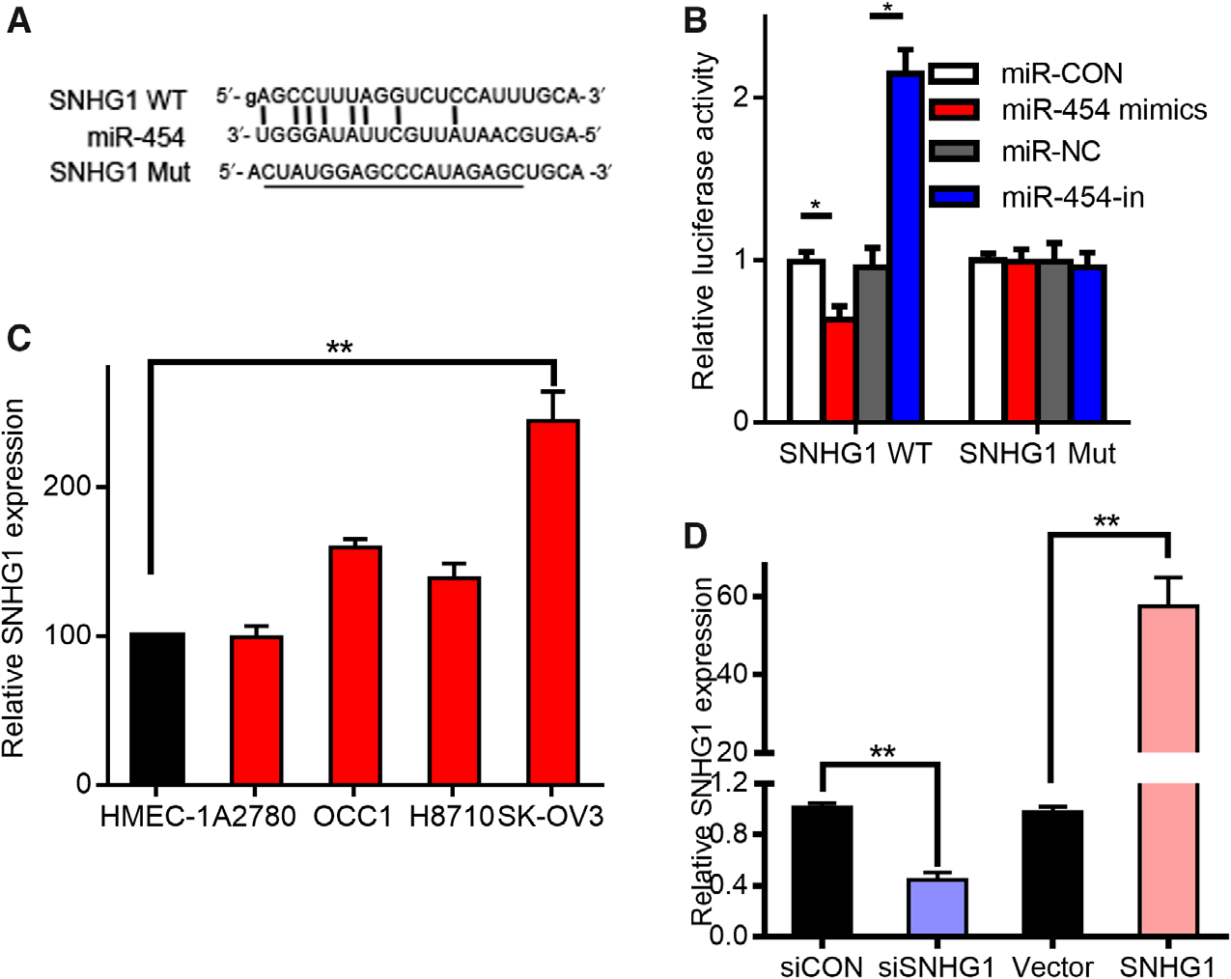
MiR‐454 is modulated by lncRNA SNHG1. (A) The miR‐454 putative and mutant regions in SNHG1 mRNA. (B) Impact of miR‐454 on the reporter genes containing wt‐SNHG1 or mut‐SNHG1 sequences. * vs Blank, *P* < 0.05. (C) Relative content of SNHG1 in OC and healthy cell lines. ** vs HMEC‐1, *P* < 0.01. (D) Relative proportion of SNHG1 in SK‐OV3 cell line subsequent to siSNHG1 transfection. ** vs control siRNA (siCON), *P* < 0.01. The relative proportion of SNHG1 in the SNHG1‐transfected A2780 cell line. Measurement data are expressed as mean ± standard deviation. Comparisons among multiple groups were analyzed using one‐way ANOVA. ** vs Blank vector, *P* < 0.01.

We investigated whether the modulation of ZEB1 by miR‐454 is regulated by lncRNA. The binding sites between miR‐454 and SNHG1 were recognized through bioinformatics. To study how SNHG1 and miR‐454 interact, we created a reporter plasmid with a predicted miR‐454 binding site on the mRNA of SNHG1, and the corresponding reporter plasmids with mutant miR‐454 binding sites (Fig. 5A). Luciferase reporter gene evaluation revealed that miR‐454 inhibited the reporter function of wt‐SNHG1 only (Fig. 5B). Next, we measured the expression of SNHG1 in healthy cells (HMEC‐1) and OC cells (A2780, OCC1, H8710 and SK‐OV3): the level of SNHG1 in healthy cells was greater than that in the OC cells. In addition, the level in SK‐OV3 cells was considerably greater relative to A2780 cells (Fig. 5C). The SNHG1 level in SK‐OV3 cells was reduced after transfection with si‐SNHG1, whereas that of A2780 cells was notably augmented after transfection with pcDNA‐SNHG1 (Fig. 5D).

### 3.6 LncRNA SNHG1 regulates OC via miR‐454

**Fig. 6:**
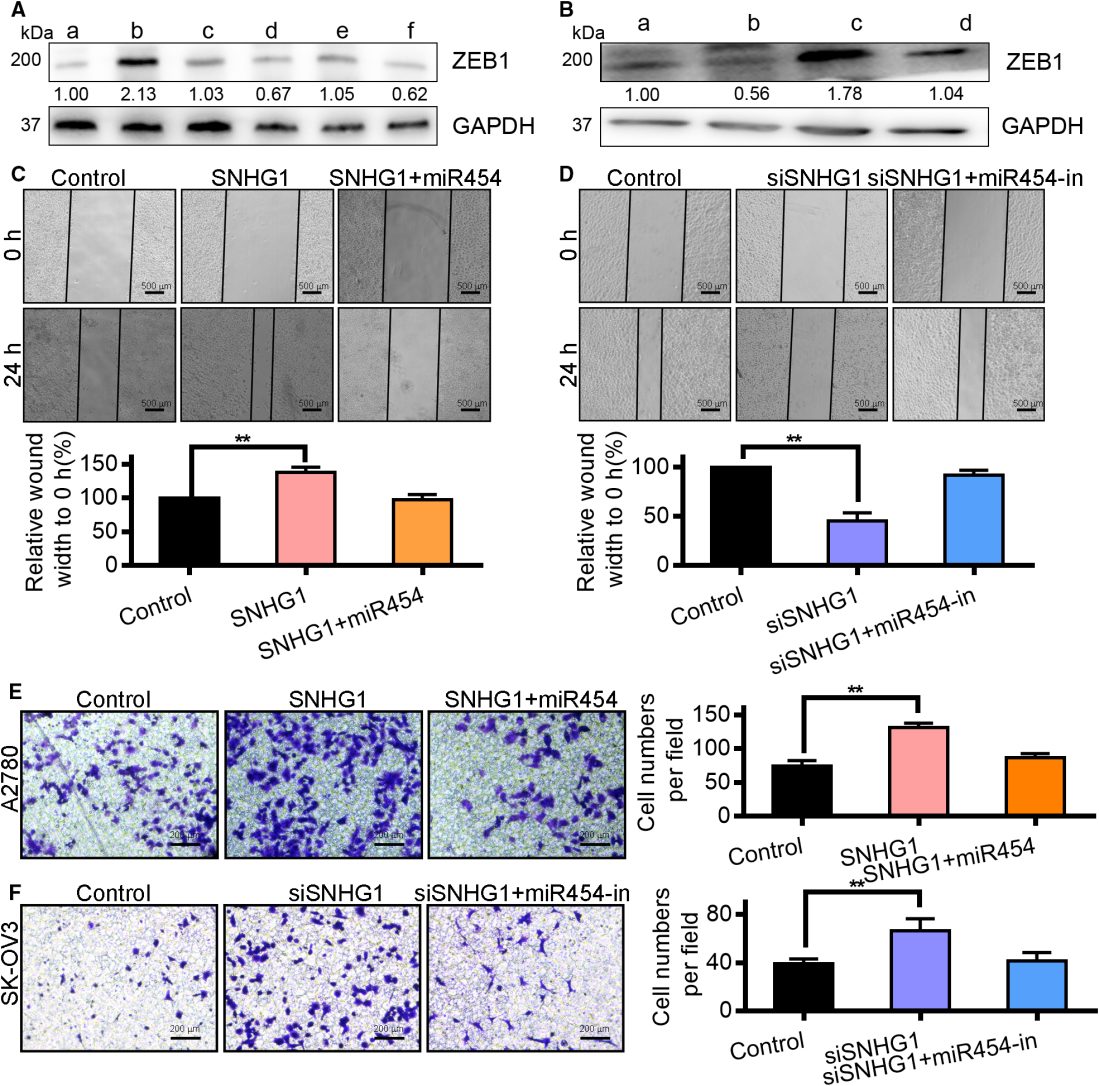
LncRNA SNHG1 regulates OC via miR‐454. (A) The control of SNHG1 on ZEB1 was mediated by miR‐454 in A2780 cell line: (a) control group; (b) pcDNA‐SNHG1 treatment group; (c) pcDNA‐SNHG1‐mut treatment group; (d) miR‐454 mimics treatment group; (e) mimics and pcDNA‐SNHG1 treatment group; (f) mimics and pcDNA‐SNHG1‐mut treatment group. (B) The control of SNHG1 on ZEB1 was mediated through miR‐454 in SK‐OV3 cell line: (a) control group; (b) si‐SNHG1 treatment group; (c) miR‐454‐in treatment group; (d) miR‐454‐in and si‐SNHG1 treatment group. (C) The regulation of SNHG1 on OC cell migration was mediated through miR‐454 in A2780 group. Scale bar: 500 μm. ** vs pcDNA‐SNHG1 group, *P* < 0.01. ** vs mimics or miR‐454‐in group, *P* < 0.01. (D) The control of SNHG1 on OC cell migration was mediated through miR‐454 in SK‐OV3 group. Scale bar: 500 μm. ** vs pcDNA‐SNHG1 group, *P* < 0.01. ** vs mimics or miR‐454‐in group, *P* < 0.01. (E) SNHG1 promoted the invasiveness of A2780 cells, whereas the transfection with miR‐454 mimic prevented the promotion. Scale bar: 200 μm. ** vs pcDNA‐SNHG1 group, *P* < 0.01. (F) Downregulation of SNHG1 arrested the invasion of SK‐OV3 cells, whereas the transfection with miR‐454‐in eliminated the inhibition. Scale bar: 200 μm. The measurement data are expressed as mean ± standard deviation. Comparisons among multiple groups were analyzed using one‐way ANOVA. ** vs si‐SNHG1 group, *P* < 0.01.

To study the relationship between lncRNA SNHG1 and miR‐454, the protein expressions of ZEB1 in A2780 cells subsequent to transfection with pcDNA‐SNHG1, pcDNA‐mut‐SNHG1, miR‐454 mimic, mimic + pcDNA‐SNHG1, and mimic + pcDNA‐mut‐SNHG1 were determined by western blotting. The results showed that the ZEB1 expressions in the miR‐454 mimic‐ and the pcDNA‐SNHG1‐group were reduced and increased, respectively, relative to the blank group. On the other hand, the negative action of miR‐454 mimic on ZEB1 was abolished by pcDNA‐SNHG1, but pcDNA‐mut‐SNHG1 had no influence on the inhibitory action of miR‐454 mimic on ZEB1 (Fig. 6A). Additionally, the protein expression of ZEB1 was reduced by si‐SNHG1 and increased by miR‐454‐in, and this effect was abolished by si‐SNHG1 (Fig. 6B). In the wound‐healing assay, pcDNA‐SNHG1 induced the migration of A2780 cells, whereas pcDNA‐SNHG1 + miR‐454 mimic decreased it. In contrast, overexpression of miR‐454 in A2780 cell line was able to abolish the weakening effects of pcDNA‐SNHG1 (Fig. 6C). The migration of cells treated with siSNHG1 was significantly decreased relative to control cells, whereas the knockdown of miR‐454 in SK‐OV3 cells was able to abolish this effect (Fig. 6D). The Transwell assay showed that invasiveness of cells treated with siSNHG1 was significantly decreased, whereas knockdown of miR‐454 abolished the enhancing effects of siSNHG1 on cell invasiveness. Additionally, pcDNA‐SNHG1 significantly induced the invasiveness of A2780 cells compared with that of control cells. Moreover, miR‐454 overexpression in A2780 cells abolished the invasiveness‐inducing effects of pcDNA‐SNHG1 (Fig. 6E,F). Thus, SNHG1 and miR‐454 were able to interact with and inhibit each other directly, and SNHG1 was able to promote OC cell migration and invasiveness by targeting miR‐454.

### 3.7 SNHG1 is essential for tumor progression *in vivo*

**Fig. 7:**
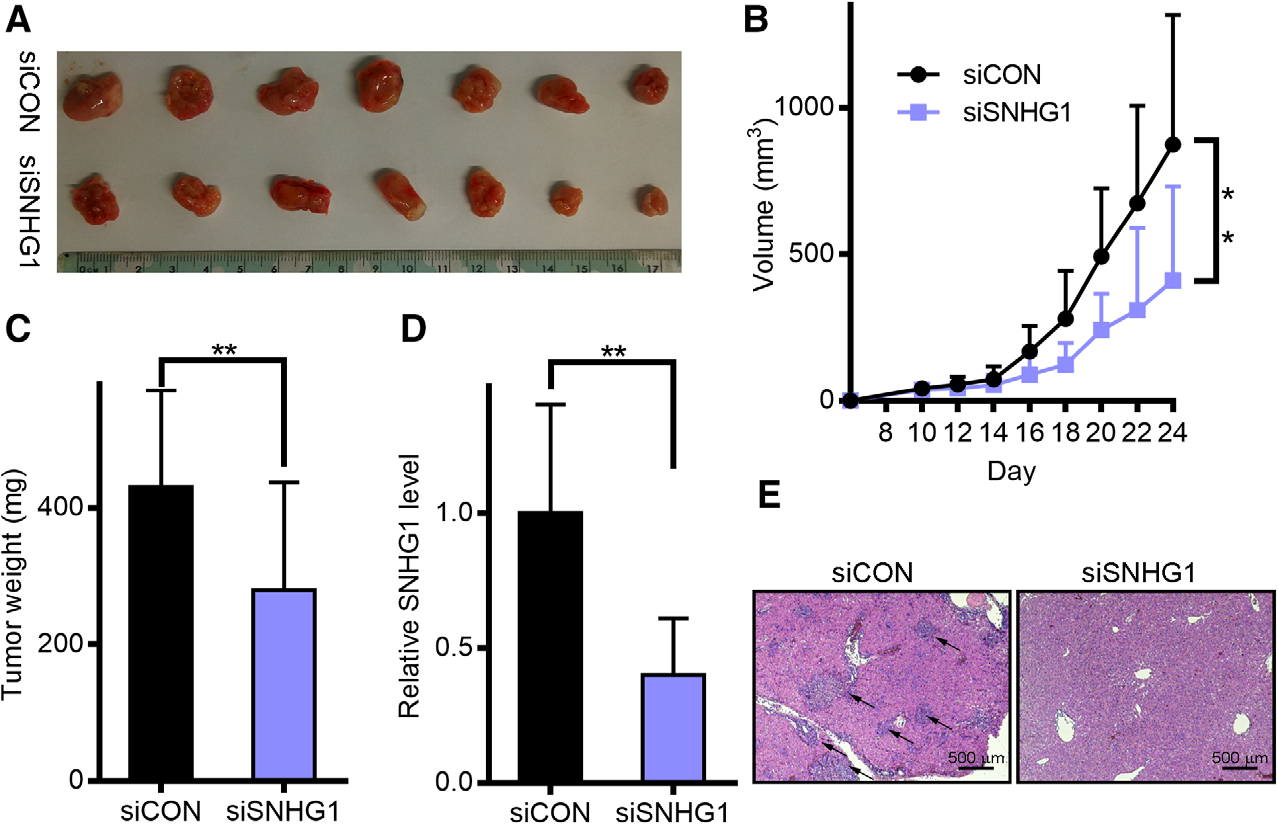
Influence of lncRNA SNHG1 knockdown on tumor proliferation *in vivo*. (A) Number of tumors excised from the mice 24 days following injection of siSNHG1‐ or shCON‐transfected A2780 cells. (B) Tumor growth curve: points denote mean (*n* = 7) and bars denote SD. (C) Tumor weights are provided as mean ± SD when excised. (D) SNHG1 expression in siSNHG1 A2780 cells relative to siCON cells, as measured by qRT‐PCR. (E) Photographs (×40) of H&E staining of the tumors. Scale bar: 500 μm. The measurement data are expressed as mean ± standard deviation. Comparisons between two groups were analyzed using non‐paired *t*‐tests. **P < 0.01.

To assess the role of SNHG1 in tumor development, siSNHG1 or control siCON‐transfected A2780 cells were administered through subcutaneous injection in mice. Similar to the *in vitro* outcomes, tumor proliferation in the siSNHG1 group was notably reduced compared with the siCON group (Fig. 7A,B). Three weeks after injection, the dimensions and weights of the tumors were decreased in the siSNHG1 group relative to the siCON group (Fig. 7A–C). The suppression of SNHG1 in the xenografted tumors from the siSNHG1 group was confirmed through qRT‐PCR analysis (Fig. 7D). Furthermore, hematoxylin and eosin staining (H&E) indicated that tumors from the siCON group displayed a greater metastasis compared with the siSNHG1 group (Fig. 7E). Our results thus indicate that SNHG1 affects OC proliferation both *in vitro* and *in vivo*.

## 4 Discussion

Tumor relapse, enhanced invasiveness and metastasis all present challenges in the prognosis and treatment of OC [1]. In spite of an improved knowledge of the genetic changes involved in OC, the development of effective treatment strategies has been restricted by the difficulty in identifying relevant biomarkers of the disease. Therefore, the identification of molecular markers of OC will be vital to a better comprehension of its biology and, eventually, for the discovery of novel pharmacological targets.

Recently, miRNA were identified to control tumor metastasis via inhibition of several target genes and have been used as cancer‐associated biomarkers [26]. Huang *et al*. [27] reported that the miR‐590‐3p was able to promote invasiveness and metastasis of OC. The miR‐143‐3p inhibits OC metastases by directly repressing transforming growth factor‐β‐activated kinase 1 [28]. We therefore set out to identify miRNA that regulate OC metastasis by analyzing miRNA expression profiles. MiR‐454 expression has been upregulated in normal ovarian tissue and cells compared with OC cells (A2780, SK‐OV3, OCCA and H8710). These observations are consistent with miR‐454 being a tumor suppressor in OC with key roles in tumor invasiveness and metastasis. MiRNA control various molecular pathways and can serve as tumor suppressors or oncogenes by targeting particular mRNA. MiR‐28‐5p can promote OC by negatively regulating tumor suppressor NEDD4 binding protein 1 (N4bp1) [29] and by promoting the progress of EMT.

Fan *et al*. [18] demonstrated that miR‐454 promoted the development, invasiveness and pro‐angiogenic activity of PDAC cells *in vitro* and lung metastasis *in vivo*. Furthermore, the proportion of miR‐454 in PDAC tissues was reduced relative to normal cells [30]. Nevertheless, Yu *et al*. [17] validated that knockdown of miR‐454 hindered cell growth, invasiveness and EMT, whereas overexpression promoted these effects in hepatocellular carcinoma. Li *et al*. reported that miR‐454 promoted the tumorigenesis of TNBC cells, and improved their migration and invasiveness. MiR‐454 inhibited radiation‐induced apoptosis in TNBC cells after ionizing radiation by regulation of caspase‐3/7 and Bcl‐2 expression [31]. That work revealed that miR‐454 content in OC tissues was reduced relative to healthy controls. Moreover, some research groups have reported miR‐454 downregulation, whereas others have shown its upregulation during cancer development. Here, we established that upregulated miR‐454 plays a significant role in the modulation of OC.

The proportion of miR‐454 in normal cells was much higher than in the OC cell lines (Fig. 1B) and tissues. Hence, we infer that miR‐454 was strongly linked to OC metastasis. Additionally, miR‐454 upregulation hindered the development, migration and invasiveness of OC cells, whereas downregulation of miR‐454 promoted these processes (Fig. 2). Overall, we infer that miR‐454 plays a crucial function in the invasiveness and metastasis of OC.

Epithelial–mesenchymal transition is a critical phenomenon that occurs during embryonic development and cancer progression [32]. This work demonstrated that the expression of EMT‐related proteins (Vimentin, ZO‐1, and N‐cadherin) in OC cells was noticeably reduced after the overexpression of miR‐454, whereas that of E‐cadherin was amplified (Fig. 3A) [32]. Thus, we infer that the upregulation of miR‐454 suppresses EMT. The levels of MMP‐2 and ‐9, which play important roles in tumor cell invasiveness and metastasis, decreased [33]. Since there are numerous signaling pathways associated with EMT [34], we tested some of these pathways and observed that the PI3K/Akt cascade is modulated by miRNA. This pathway controls survival signals, thwarts the apoptosis of OC cells, and supports oncogenesis [35, 36, 37].

Zinc finger E‐box‐binding homeobox 1 is capable of suppressing tumors and inhibiting the activation of PI3K/Akt cascade [38]. It can activate Akt kinases, which are involved in cell growth and invasiveness [39]. Herein, we ascertained that ZEB1 is directly targeted by miR‐454 such that its overexpression suppresses the expression of ZEB1, whereas the silencing enhances its expression (Fig. 3). We inferred that upregulated miR‐454 diminished the proportion of ZEB1, which activated PI3K/Akt signaling pathway as well as the metastasis of OC. According to our present study, we determined a putative mechanism by which lncRNA SNHG1 may play a novel oncogenic role in cell growth by modulating PI3K/Akt signaling.

Of late, numerous studies have established that miRNA are under the control of lncRNA [40]. We speculated that miR‐454 inhibits OC metastasis in a manner modulated by lncRNA. Bioinformatics analysis revealed that miR‐454 has binding sites with SNHG1 (Fig. 5A). SNHG1 plays key roles in several tumors such as testicular germ cell tumors and OC [41, 42]. For the first time, we have shown that knockdown of SNHG1 upregulated miR‐454 expression and miR‐454 knockdown enhanced SNHG1 expression (Fig. 6). In addition, a higher expression miR‐454 diminished the ZEB1 levels, whereas the further transfection with pcDNA‐SNHG1 eliminated this effect. The development, migration and invasiveness of OC cells after SNHG1 downregulation were notably reduced relative to those of control cells. PcDNA‐SNHG1 induced the metastasis and invasiveness of OC cells, whereas this effect was abolished following miR‐454 mimic‐transfection. Nevertheless, transfection with the inhibitor eliminated the positive effect of siSNHG1 on OC cell migration and invasiveness. Therefore, we demonstrated that miR‐454 and SNHG1 are vital for the progression of OC.

## 5 Conclusions

The present work not only established the vital function of SNHG1/miR‐454/ZEB1 signaling cascade in OC pathogenesis but also indicated prospective roles for both miR‐454 and SNHG1 in the prognosis and therapeutic intervention of OC.

## Conflict of interest

The authors declare no conflict of interest.

## Author contributions

JR conceived and supervised the study; JR and YW designed the experiments. JR, YW, BZ, SB, YY, HK WM, JZ, YG and RL performed experiments. BH provided new tools and reagents. JR, YW, BZ, SB, YY, HK WM, YG and BH analyzed the data. JR and YW wrote the manuscript. JR, WM, JZ, BH and XZ revised the manuscript. All authors read and approved the final version of this submission.

## Ethics approval and consent to participate

This study was approved by the Ethics Committees of Xi’an Jiaotong University. Informed consent was obtained from each patient, including consent for their samples to be taken and used for research purposes before surgery.

## Supporting information


**Table S1.** Sequences of miRNA.
**Table S2.** Sequences of primers for qRT‐PCR.
