# Supplementary material for: Long non‐coding RNA SNHG1 stimulates ovarian cancer progression by modulating expression of miR‐454 and ZEB1
Source: Mol Oncol. 2021 Mar 19;15(5):1584–96. doi: 10.1002/1878-0261.12932 (PMC8096788; doi:10.1002/1878-0261.12932)
Supplement: Supplementary file 1 — Table S1. Sequences of miRNA. Table S2. Sequences of primers for qRT‐PCR. [file MOL2-15-1584-s001.docx]

Supplementary Table 1: Sequences of miRNA

| \| Name \| \| --- \| | Sequences (5′-3′) |
| --- | --- | --- |
| miR-454 mimics | UAGUGCAAUAUUGCUUAUAGGGU |
| miR-454-in | ACCCUAUAAGCAAUAUUGCACUA |
| miR-NC | GUCUCCUAACUUCAACUAGCUG |

Supplementary Table 2: Sequences of primers for qRT-PCR

| \| Primers for qRT-PCR \| \| --- \| | Sequences (5′-3′) |
| --- | --- | --- |
| miR-454 | TAGTGCAATATTGCTTATAGGG |
| U6 snRNA (Forward) | TGCGGGTGCTCGCTTCGGCAGC |
| SNHG1 (Forward) | AGGCTGAAGTTACAGGTC |
| SNHG1 (Reverse) | TTGGCTCCCAGTGTCTTA |
| GAPDH (Forward) | TGCACCACCAACTGCTTAGC |
| GAPDH (Reverse) | GGCATGGACTGTGGTCATGAG |
